# Comparison of volumetric-modulated arc therapy using simultaneous integrated boosts (SIB-VMAT) of 45 Gy/55 Gy in 25 fractions with conventional radiotherapy in preoperative chemoradiation for rectal cancers: a propensity score case-matched analysis

**DOI:** 10.1186/s13014-017-0894-9

**Published:** 2017-09-21

**Authors:** Hideomi Yamashita, Soichiro Ishihara, Hiroaki Nozawa, Kazushige Kawai, Tomomichi Kiyomatsu, Kae Okuma, Osamu Abe, Toshiaki Watanabe, Keiichi Nakagawa

**Affiliations:** 1 Department of Radiology, University of Tokyo Hospital, 7-3-1, Hongo, Bunkyo-ku, Tokyo, 113-8655 Japan; 2 Department of Surgical Oncology, University of Tokyo Hospital, Bunkyo-ku, Japan

**Keywords:** Preoperative radiotherapy, Rectal cancer, Intensity-modulated radiotherapy, Image-guided radiotherapy, Simultaneous integrated boost

## Abstract

**Background and purpose:**

The aim of this retrospective study was to compare volumetric-modulated arc therapy using simultaneous integrated boosts (SIB-VMAT) of 45 Gy/55 Gy in 25 fractions with three-dimensional conformal radiotherapy (3D–CRT) in preoperative chemoradiation for rectal cancers.

**Methods and materials:**

In the propensity score-matching analysis of 1:2, we selected 60 patients from the SIB-VMAT group and 120patients from the 3D–CRT group matched pairings out of 145 patients between 2005 and 2015. The regimen of concurrent combined chemotherapy was oral uracil/tegafur plus leucovorin with/without irinotecan.

**Results:**

There were no significant differences between the two groups, in pathological complete response rates (pCR) (11% in the 3D–CRT group vs. 17% in the SIB-VMAT group, *P* = 0.39), pathological response rates (44% vs. 60%, *P* = 0.77), disease-free survival (*P* = 0.32), or local control (*P* = 0.52). The SIB-VMAT method marginally improved the rate of pathological grade 2–3 effects and the OS was significantly better in patients with grade 2–3 effects. Recurrence was seen in 36 patients (30%) in the 3D–CRT group and 19 patients (32%) in the SIB-VMAT group. The first distant recurrence site in the SIB-VMAT group was liver in 6 patients and lung in 8 patients. The obvious radiation-induced late toxicity in the SIB-VMAT group was recto-vesical fistula in two patients.

**Conclusions:**

The SIB-VMAT may be a promising method for preoperative CRT of rectal cancer.

## Introduction

Combination treatment composed of surgery and 5-fluorouracil-based chemotherapy concurrently combined with whole pelvis irradiation is suggested for most patients with stage II-III rectal cancer [1–4]. The pre- or post-operative pelvis radiation therapy (RT) for these patients also continues to improve [5].

Many randomized phase III studies have estimated the efficacy of adding chemotherapy to preoperative RT in rectal cancer [6]. The estimated advantages of adding chemotherapy to RT include an increase in local RT sensitivity and a better control of the systemic disease by eradicating microscopic distant metastases. Additionally, neoadjuvant chemoradiation therapy (CRT) provides the possibility of improving the proportion of patients with sphincter preservation and/or pathologic complete response (pCR).

If image-guided radiation therapy (IGRT) and volumetric modulated arc therapy (VMAT) are used, normal tissue damage is less likely to occur, and the setup margins can be minimized; in addition, in another advantage of this technique, it may be possible to deliver a simultaneous integrated radiation boost (SIB) to the gross tumor volume [7].

The aim of this retrospective study was to compare SIB-VMAT of 45 Gy/55 Gy in 25 fractions with conventional radiotherapy in preoperative chemoradiation of rectal cancers.

## Methods and materials

Patients had to present with histopathologically confirmed rectal adenocarcinoma involving the middle or lower third of the rectum (below the peritoneal reflection) and evidence of T3/T4, any N, M0 disease on MRI or endoluminal ultrasound and normal liver, renal, and bone marrow functions. Patients with unresectable metastatic disease at diagnosis were excluded. Other exclusion criteria were as follows: prior chemotherapy for rectal cancer or any prior pelvic irradiation; severe heart disease, uncontrolled infection or metabolic disorders; or severe neurologic impairment or inflammatory bowel disease.

Three-dimensional conformal radiotherapy (3D–CRT) of 50.4 Gy was performed in 145 patients from February 2005 to December 2011 and from April 2014 to September 2015. SIB-VMAT of 45 Gy/55 Gy in 25 fractions was administered to 60 consecutive patients between January 2012 and March 2014.

### Radiotherapy technique

#### 3D–CRT group

RT began on the first day of chemotherapy and was administered 5 times per week with a daily fraction of 1.8 Gy. Initially, the entire pelvis was treated with 3- or 4-field techniques to 50.4 Gy in a supine position using a 10 MV X-ray accelerator. The irradiation field wasn’t changed on the way as one series. The clinical target volume (CTV) included the entire pelvic cavity, the anal canal, the primary tumor, mesorectal and presacral lymph nodes, nodes along the internal iliac artery, lumbar nodes up to the level of the lower border of the fifth lumbar vertebra, and nodes at the obturator foramen. The superior border of the entire pelvis was placed at the bifurcation of internal and external iliac arteries. Uniform planning target volume (PTV) margins of 5 mm in the lateral, the anteroposterior, and the craniocaudal directions were applied. A 3-D conformal technique has been used with planning CT. The same machine had been used for SIB-VMAT groups.

#### SIB-VMAT group

**Fig. 1:**
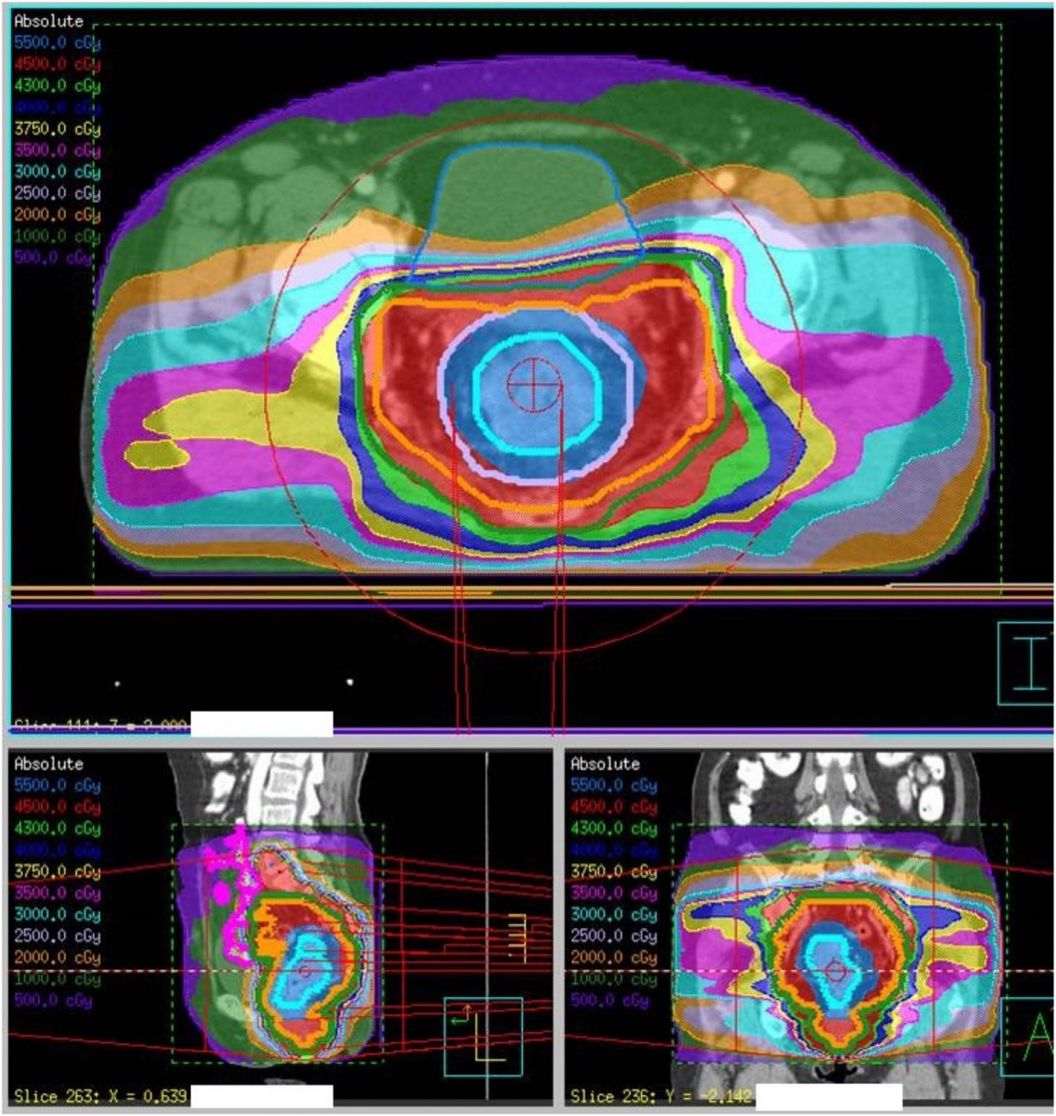
A dose distribution in the SIB-VMAT group. (sky blue line = GTV, purple = PTV1, orange = CTV, green = PTV2, pink = small bowel)

Preoperative RT was carried out with IMRT–IGRT using the Elekta Synergy linac with Agility collimating device. The details of the SIB-VMAT method was described in our previous report [8]. All patients received a dose of 45 Gy in daily fractions of 1.8 Gy to the primary tumor, the mesorectum, and draining lymph nodes. The 60 patients of the SIB-VMAT group received an SIB of 0.4 Gy per day on the primary tumor, up to a total dose of 10 Gy (Fig. 1). We tried to minimize the volume of small bowel receiving 15 Gy or greater (V15-SB <150 ml) and a mean bladder dose <21 Gy. Before each treatment session, patients underwent daily image guidance using the integrated kV-CT modality and were repositioned after coregistration of these images with the planning kV-CT scan. Patients were advised to drink 250 ml of water 60 min prior to the planning CT and prior to every treatment session.

### Chemotherapy

The most common concurrent chemotherapy regimen (89%) was 5-days-on/2-days-off oral uracil/tegafur (UFT) of 300 mg/m^2^/day plus leucovorin (LV) of 75 mg/body/day. The second most common concurrent chemotherapy regimen (11%) was UFT, LV, and irinotecan (CPT-11). Although CPT-11 is not a standard chemotherapy agent in the management of rectal cancer as a preoperative setting, it has been used for a limited time in preoperative CRT.

### Surgical technique

A variety of surgical approaches, depending on the location and extent of disease, were used to treat primary rectal cancer lesions. These methods include invasive procedures such as transabdominal resection as low anterior resection (LAR), intersphincteric resection (ISR), and abdominoperineal resection (APR) [9, 10]. All surgeries were performed by colorectal specialists. The interval between radiotherapy and surgery was planned as not less than 4 weeks. In most patients, surgery has been performed around the 8th week after completing CRT.

### Pathological analysis

Analysis of the surgical specimens included determination of the following parameters: a) histologic type of the tumor; b) degree of extension of the tumor through the rectal wall; c) nodal involvement; and d) status of proximal and distal margins. Post-CRT histological tumor regression was graded according to the seventh edition of the Japanese General Rules for Clinical and Pathological Studies on Carcinoma of the Colorectum: Grade 0, neither necrosis nor regression change; Grade 1a, >2/3 viable residual tumor cells; Grade 1b, approximately 1/3 to 2/3 viable residual tumor cells; Grade 2, <1/3 viable tumor cells; and Grade 3, no viable residual tumor cells [11]. In this study, both grades 1a and 1b were classified together as grade 1 in the same manner as many previous studies [7, 12, 6].

### Statistical analysis

The primary endpoint of this study is the comparison of pCR rate in the surgery specimen. The secondary endpoints are the comparison of disease-free survival, local control, and late toxicity.

We applied a 1:2 propensity score matching (PSM) ratio to minimize such differences as age, sex, pre-CRT clinical T stage, N stage and pre-CRT serum carcinoembryonic antigen (CEA) value, with/without combined CPT-11, with /without anal canal invasion, and pathological type. The surgical technique and interval between radiotherapy and surgery were not included as matching factors, because we considered that these factors could be influenced greatly by RT method such as 3D–CRT or SIB-VMAT and these were a part of treatment effects due to differences in RT method. In the PSM analysis, we selected 60 patients from the SIB-VMAT group with 120 matched pairings of the 3D–CRT group using a randomized nearest-neighbor algorithm. A 1:2 matching was performed to minimize the effects of small sample size on PSM and because there was a wide difference in the number of patients in each group before PSM (60 and 145 patients). We used “R” as statistics analysis software (http://www.R-project.org/).

With regard to comparison of the background factors of the two groups, the continuous variable examines the difference of means by an unpaired t-test, and a chi square test was performed for the nominal variable. Survival periods were calculated from the start of 3D–CRT or SIB-VMAT. The survival functions were estimated with the Kaplan-Meier method estimator, and log-rank tests were used to compare the survival distributions. The borderline-difference was defined as *P* < 0.1.

## Results

With a median time of 8.1 weeks (range; 4.1–14.5 weeks) after completion of preoperative RT, LAR, ISR, APR, and other operative methods (Hartmann’s operation or pelvic evisceration) were performed in 70, 18, 9, and 3% of patients, respectively. In 96% patients, the interval between preoperative RT end and surgery was more than 5 weeks and in 37% patients, it was more than 9 weeks. There was no significant difference by two groups about this interval by an unpaired t-test (*P* = 0.69). In one patient in the 3D–CRT group, local resection, which was not standard treatment, had been performed repeatedly at his wish. Since it was thought that whether pCR or not of the primary endpoint can be evaluated, this patient was not excluded.

The clinicopathological characteristics were balanced and evenly distributed between the groups (all *P* > 0.1) except in those with/without anal canal invasion (*P* = 0.0040) (Table 1). The median follow-up time was 44.7 months in all 180 patients, 38.1 months (range; 16.6–50.5 months) in the SIB-VMAT group, and 59.2 months (range; 8.6–131.7 months) in the 3D–CRT group. In all 180 patients, there was no significant difference in disease-free survival (DFS), overall survival (OS), and local control (LC) between an radiation-surgery interval ≤ 7 weeks vs. >7 weeks (*P* = 0.42, 0.55, and 0.56) or between ≤9 weeks vs. >9 weeks (*P* = 0.54, 0.59, and 0.19), respectively.

**Table 1:** Clinicopathological characteristics after PSM

Factors	3D–CRT		SIB-VMAT		*p* value
	N	Rate	N	Rate	
Total	120		60		
Age					
Median	64 y.o.		66 y.o.		0.81
Range	32–83 y.o.		44–88 y.o.		
Sex					
Female	41	34%	17	28%	0.43
Male	79	66%	43	72%	
Clinical T stage					
cT2	10	8%	3	5%	0.23
cT3	101	84%	48	80%	
cT4	9	8%	9	15%	
Clinical N stage					
cN0	67	56%	25	42%	0.102
cN1–2	53	44%	35	58%	
cN0	67	56%	25	42%	0.053
cN1	29	24%	25	42%	
cN2	24	20%	10	17%	
Clinical stage					
c-II	65	54%	25	42%	0.11
c-III	55	46%	35	58%	
Distance from AV					
Median	5 cm		4 cm		0.56
Range	0–12 cm		0–12 cm		
Serum CEA value					
Median	6.4		7.2		0.78
Range	1.2–231.8		1.5–93.1		
Combined CPT-11					
With	14	12%	6	10%	0.74
Without	106	88%	54	90%	
Location of primary tumor					
Upper rectum	36	30%	11	18%	0.0053
Lower rectum	73	61%	33	55%	
Anal canal	11	9%	16	27%	
Histopathological type					
Well-differentiated					
adenocarcinoma	65	54%	37	62%	0.34
Others	55	46%	23	38%	

The operative method LAR/ISR with sphincter preservation was performed in 95 patients (79%) in the 3D–CRT group and 53 patients (88%) in the SIB-VMAT group (*P* = 0.13). In both groups, there was no significant difference in either pCR rate (11% in the 3D–CRT group and 17% in the SIB-VMAT group, *P* = 0.39) or pathological response rate (pRR) of effective grades 2 plus 3 rates (44% and 60%, respectively, *P* = 0.065). The number of patients with pathological responses grades 0 / 1a / 1b / 2 / 3 were 0% / 21% / 35% / 33% / 11% in the 3D–CRT group and 3% / 11% / 29% / 43% / 17% in the SIB-VMAT group, respectively (*P* = 0.102). The mean / median interval between radiotherapy and surgery was 13.9 / 13.7 weeks of the patients with grades 2–3 and 14.0 / 13.7 weeks of those with grades 0–1. We could not adjust for numbers with or witho + ut anal canal invasion even after PS matching, and there were significantly more patients with anal canal invasion in the SIB-VMAT group (27% vs. 9%, *P* = 0.0040). The radial margin (RM) positive ratewas 0% in the SIB-VMAT group and 1.7% (two patients) in the 3D–CRT group.

**Fig. 2:**
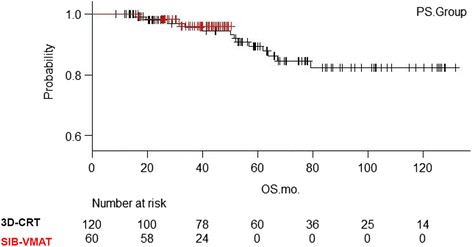
Kaplan-Meier overall survival curves for the matched groups. Red line = SIB-VMAT group. Black line = 3D–CRT group

**Fig. 3:**
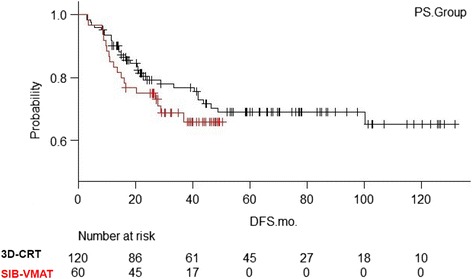
Kaplan-Meier disease-free survival curves for the matched groups. Red line = SIB-VMAT group. Black line = 3D–CRT group

**Fig. 4:**
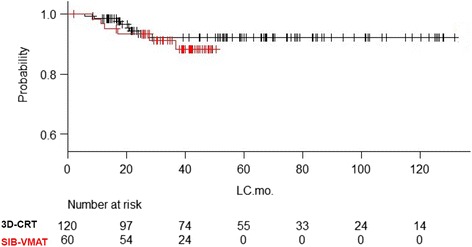
Kaplan-Meier local control curves for the matched groups. Red line = SIB-VMAT group. Black line = 3D–CRT group

The Kaplan-Meiersurvival curves for the matched groups are shown in Fig. 2. In the survival analysis of the matched 180 patients, the 2-y and 3-y OS was 98.1% (95% CI; 92.6–99.5%) and 95.7% (95%CI; 88.9–98.4%), respectively, for 3D–CRT patients and 98.3% (95%CI; 88.6–99.38%) and 96.0% (95%CI; 94.5–99.0%), respectively, for SIB-VMAT patients (*P* = 0.77). The 2-y and 3-y DFS (Fig. 3) was 80.3% (95% CI; 71.6–86.6%) and 76.7% (95%CI; 67.4–83.7%), respectively, for 3D–CRT patients and 75.0% (95%CI; 61.9–84.1%) and 68.9% (95%CI; 55.1–79.2%), respectively, for SIB-VMAT patients (*P* = 0.32). Since both the more recent cases from 2014 to 2015 and the older cases were included in the 3D–CRT group, there are more censored cases in the first half of the curve. The 2-y and 3-y LC (Fig. 4) was 94.4% (95% CI; 87.8–97.4%) and 92.0% (95%CI; 84.6–96.0%), respectively, for 3D–CRT patients and 93.2% (95%CI; 82.9–97.4%) and 91.1% (95%CI; 79.8–96.2%), respectively, for SIB-VMAT patients (*P* = 0.52). Pelvic disseminated recurrence was not included as a local recurrence.

The 3-y OS was 97.6% vs. 93.9% in patients less than vs. not less than 65 years old (log-rank *P* = 0.86); 91.8% vs. 96.7% in patients with less than vs. not less than 20 ng/mL of pre-CRT serum CEA (*P* = 0.16); 95.8% vs. 95.8% between less than vs. not less than 3/4-circumferential (*P* = 0.41); 98.5% vs. 93.3% between cN0 vs. cN1–2 (*P* = 0.50); 100% vs. 95.1% between patients with vs. without CPT-11 (*P* = 0.86); 100% vs. 95.5% between cT1–2 vs. cT3–4 (*P* = 0.19); 100% vs. 95.3% between those with effective grade 3 vs. the others (*P* = 0.21); 94.6% vs. 98.8% between those with effective grade 1 vs. grades 2–3 (*P* = 0.0067, 84.5% vs. 95.8% in 5-y OS); 97.7% vs. 92.8% between those with well-differentiated adenocarcinoma vs. the others (*P* = 0.82); 95.8% vs. 95.9% between those with vs. without anal canal invasion (*P* = 0.26); and 100% vs. 93.9% between female vs. male patients (*P* = 0.23).

Recurrence was seen in 36 patients (30%) in the 3D–CRT group and 19 patients (32%) in the SIB-VMAT group. Local and distant recurrence occurred in 10 patients (8%) and 26 patients (22%) in the 3D–CRT group and 6 patients (10%) and 13 patients (22%) in the SIB-VMAT group. The first distant recurrence in the SIB-VMAT group was liver metastasis in six patients and lung metastasis in eight patients.

The acute toxicity in the SIB-VMAT group was fatigue in 26%/63%/12% of grades 0/1/2, respectively, appetite loss in 34%/36%/30%, abdominal pain in 26%/60%/15%, anal pain in 4%/17%/79%, nausea in 53%/30%/17%, diarrhea in 19%/38%/42%, and urinary tract symptom in 6%/45%/49%. No grade 3–5 acute toxicity was seen. The late toxicity in the SIB-VMAT group was only radiation-induced recto-vesical fistula in two patients at 15 and 22 months after CRT, respectively.

The acute toxicity in the 3D–CRT group was grade 1–2 diarrhea in 83% and grade 1–2 anal pain in 75%. No grade 3–5 acute toxicity was seen. The late toxicity in the 3D–CRT group was radiation-induced recto-vesical fistula in one patient at 6 months after CRT and recto-vaginal fistula in another patient. Additionally, pelvic suppuration, probably caused by CRT, was seen in one patient.

## Discussion

This is a study on rectal cancer patients treated with preoperative chemoradiation with a V-MAT SIB technique, compared with an historical group of patients treated with 3D technique at a single institution. No statistically significant difference could be found for pCR and pRR, DFS and LC. Late toxicity seemed to be acceptable. Although interval between preoperative RT and surgery has been reported an important prognostic factor in treatment of rectal cancer [13–16], there was no significant difference between both groups in the present study.

The addition of chemotherapy to preoperative radiotherapy has been established as the standard of care for patients with cT3–4 rectal cancer. Current strategies in preoperative treatment of rectal cancer have been based mainly on the combination of oxaliplatin and molecular targeted drugs plus RT in order to improve the pCR rate and to decrease the high incidence of distant recurrences. There have now been seven major trials on oxaliplatin [17–23] and two of those demonstrated a benefit for oxaliplatin regarding DFS [20, 23]. Meta-analyses have also demonstrated this benefit [24, 25]. In patients receiving neoadjuvant treatment, CRM positivity is one of the strongest predictive factors of local recurrence [26]. In this study, CRM positivity or negativity in the surgical specimens were not adjusted between the two groups because only pre-CRT factors were adjusted.

About 20% of patients showed a pCR after preoperative treatment [27, 28]. Some reports have claimed that patients with pCR improve survival compared with those with intermediate or poor regression after preoperative CRT in rectal cancer [12, 6, 29]. In the present study, however, the pathologically determined response to preoperative CRT was not correlated with long-term prognosis, and the 3-year OS was 100 and 96% for patients with complete response and the others, respectively (*P* = 0.21), though the OS of patients with effective grades 2–3 was significantly better than those with grade 1 (*P* = 0.0067). Additionally, since there was a borderline-difference in effective grades 2 plus 3 rates (60% in the SIB-VMAT group vs. 44% in the 3D–CRT group, *P* = 0.065), if the more patients are compared, the SIB-VMAT method may lead to the benefit of survival.

The 3D–CRT group contains the patients in older time from 2005 and we think that a difference in the LAR/ISR rate is mainly related to the period, not to the effect of CRT. Additionally, the reason that we could not adjust for the presence or absence of anal canal invasion was likely because cases with anal canal invasion already comprised a significant proportion of the 27% in the SIB-VMAT group, and in the 3D–CRT group the invasion did not reach this value even if a lot of cases with invasion were selected.

In the present study, the 3-year LC, DFS, and OS in the SIB-VMAT group were 91, 69, and 96%, respectively. It seems that our results were not inferior to the previous other studies [6, 30–32].

Patients with locally advanced rectal cancer receive preoperative CRT as the standard of care, producing a pCR in 10–20% and a complete clinical response in 20–30% of patients [33]. In the phase II SHOGUN trial that our institution took part in, the pCR rate in 45 patients was very high, 27.3%, after preoperative CRT with S-1 plus oxaliplatin plus radiotherapy with 50.4 Gy in 28 fractions for clinical stage T3 or T4 (any N, M0) locally advanced rectal carcinoma [34].

Treatment-related toxicity was decreased by the creation of steep dose gradients and reduction of the CTV to the PTV margin, and the implementation of IMRT and IGRT results in a minimization of the irradiated volume of the surrounding healthy tissues such as the small bowel and bladder. In the present study, 2 out of 60 patients (3.3%) in the SIB-VMAT group experienced late grade 3–4 GI toxicity but no severe genitourinary (GU) toxicity. This rate is similar in the previous reports and is well-tolerated [31, 32, 35–39].

A larger volume of normal tissue will be exposed to low-dose radiation by VMAT. The resulting steep dose gradients of VMAT decrease the volume of normal tissue exposed to high doses. The effect of both factors on the incidence of radiation-induced second malignancies is still left open [40, 41]. For rectal cancer patients receiving short-course preoperative RT, the Swedish Rectal Cancer Trial and the Dutch TME trial reported 11.7 and 14% secondary cancers after a median follow-up time of 14 years and 12 years, respectively [42, 43].

The majority of RCTs on the addition of oxaliplatin to standard CRT reported almost the same difference in pCR as in this study and, in all but one study, the difference was not significant [17–19]. In this study, the significantly higher number of patients with anal canal infiltration (27% vs. 9%) in the VMAT-SIB group might have negatively influenced tumor response as well as DFS and could explain, at least in part, the lack of a pCR difference between the two RT modalities, since rectal adenocarcinoma with infiltration of the anal canal is generally more aggressive tumors. The median follow-up was significantly longer in the 3D–CRT arm compared to that in the VMAT-SIB arm, and this difference automatically generates a major statistical bias as a limitation of this study. Additionally, the heterogeneous chemotherapy regimens constitute a limitation of the study.

## Conclusion

Although the follow-up time in the SIB-VMAT group was shorter than that in the 3D–CRT group, the rate of pCR did not increase significantly even when using the SIB-VMAT method. The SIB-VMAT method marginally improved the rate of pathological grade 2–3 effects and the OS was significantly better in patients with grade 2–3 effects. The SIB-VMAT may be a promising method for preoperative CRT of rectal cancer.

## Abbreviations

3D–CRT: Three-dimensional conformal radiotherapy
APR: Abdominoperineal resection
CEA: carcinoembryonic antigen
CPT-11: Irinotecan
CRT: Chemoradiation therapy
CTV: clinical target volume
DFS: Disease-free survival
GU: genitourinary
IGRT: Image-guided radiation therapy
ISR: Intersphincteric resection
LAR: Low anterior resection
LC: Local control
LV: Leucovorin
OS: Overall survival
PCR: Pathologic complete response
pRR: Pathological response rate
PSM: Propensity score matching
PTV: Panning target volume
RM: Radial margin
RT: Radiation therapy
SIB: Simultaneous integrated radiation boost
UFT: Oral uracil/tegafur
VMAT: Volumetric modulated arc therapy
